# The Use of Therapeutic Peptides in Combination with Full-Thickness Skin Columns to Improve Healing of Excisional Wounds

**DOI:** 10.3390/bioengineering12080856

**Published:** 2025-08-09

**Authors:** Anders H. Carlsson, Ira M. Herman, Sean Christy, David Larson, Rodney K. Chan, Thomas N. Darling, Kristo Nuutila

**Affiliations:** 1 Metis Foundation, San Antonio, TX 78216, USA; carlsson@metisfoundationusa.org (A.H.C.); rodneykchan@gmail.com (R.K.C.); 2 Department of Developmental, Molecular, and Chemical Biology, Tufts University School of Medicine, Boston, MA 02111, USA; ira.herman@tufts.edu; 3 Center for Innovations in Wound Healing Research, Tufts University School of Medicine, Boston, MA 02111, USA; 4 Tissue Health Plus, Inc., Fort Worth, TX 76102, USA; 5 United States Army Institute of Surgical Research, Joint Base San Antonio-Fort Sam Houston, San Antonio, TX 78234, USA; sean.e.christy.ctr@health.mil; 6 The Department of Surgery, University of Texas Health, San Antonio, TX 78229, USA; larsond2@uthscsa.edu; 7 Department of Dermatology, Uniformed Services University, Bethesda, MD 20814, USA; thomas.darling@usuhs.edu

**Keywords:** full-thickness skin column, therapeutic peptide, TSN6, wound healing

## Abstract

Split-thickness skin grafting (STSG) is the standard of care for skin replacement therapy. While STSG is a well-established technique, it has several limitations at both the donor and recipient sites. Full-thickness skin column (FTSC) grafting is an alternative approach that involves the orthogonal harvesting of small skin columns containing the epidermis, dermis, and associated skin appendages. Peptides have been shown to promote wound repair through various reparative and regenerative mechanisms. In this study, we aimed to evaluate the extent to which FTSCs and the matrix-derived peptide TSN6, individually or in combination, influenced the rate and quality of healing, as assessed by metrics such as epithelialization, epithelial thickness, and the presence of adnexal structures. TSN6 peptide and its scrambled form was synthetized in a laboratory and mixed with a carboxymethylcellulose (CMC) hydrogel. Up to 16 standardized full-thickness excisional wounds (∅ 5 cm) were created on the dorsum of two anesthetized pigs. FTSC biopsies (∅ 1.5 mm) were harvested from donor sites located on the rump of the pig at a ratio of up to eight 1.5 mm-diameter skin columns/1 cm^2^. Subsequently, the wounds were randomized to receive either (1) FTSC + TSN6, (2) FTSC + scrambled peptide, (3) FTSC alone, and (4) blank CMC hydrogel. Healing was monitored for 14 or 28 days. After euthanasia, the wounds were excised and processed for histology. Additionally, non-invasive imaging systems were utilized to assess wound healing. By day 14, wounds treated with FTSC or FTSC + TSN6 were significantly more re-epithelialized compared to those treated with blank CMC hydrogel. By day 28, all FTSC-transplanted wounds were fully re-epithelialized. Notably, wounds treated with FTSC + TSN6 exhibited improved healing quality, characterized by a thicker neo-epidermis and increased rete ridges at day 28 post-transplantation. All FTSC-transplanted wounds healed better than the untransplanted controls. The TSN6 peptide further improved healing quality when applied in combination with FTSCs, particularly by enhancing epidermal maturation.

## 1. Introduction

Split-thickness skin grafting (STSG) is the standard of care for skin replacement therapy. In STSG, a thin layer of autologous skin, comprising the epidermis and a portion of the dermis, is harvested using a dermatome. Before grafting, the STSG is commonly meshed at a 1:2 to 1:4 ratio to expand coverage [1,2]. Although STSG is a well-established technique, it has several limitations at both the donor and recipient sites. In cases of extensive burns, donor skin may be insufficient. Donor sites are often painful and prone to delayed healing, infection, and scarring. At the recipient site, complications may include graft failure, infection, and cosmetic issues such as hypopigmentation and scarring [3]. Additionally, since STSG contains only part of the upper dermis, it lacks adnexal structures such as hair follicles and glands. As a result, the grafted skin does not fully restore functions such as durability, thermoregulation, hydration, and lubrication [4].

Several alternative techniques to replace or complement autologous STSGs have been explored over the years [5,6,7]. One promising approach is fractional autologous skin grafting using full-thickness skin columns (FTSCs). FTSCs are harvested orthogonally by taking numerous individual full-thickness skin columns that include the epidermis, dermis, and skin appendages [8]. The FTSC donor sites are areas of small full-thickness wounds that heal fast with minimal scarring. Importantly, harvesting FTSC leaves the majority of the skin donor site, including pain-sensing nociceptors, intact [9]. Consequently, FTSC donor sites are much less painful than their STSG counterparts [10]. The reduced donor site pain and the full-thickness nature of the grafts make this technology an attractive alternative to STSG.

Peptides are functional molecules made of specific amino acid sequences that play important roles in many biological processes, including wound healing. Thousands of distinct peptides exist, each with unique biological functions [11]. They are classified as either endogenous or exogenous. Endogenous peptides are produced by various cells, such as fibroblasts, neural cells, and immune cells, or by glands like the pituitary and adrenal glands. In contrast, exogenous peptides enter the body from external sources, including food, dietary supplements, and medications. For therapeutic applications, peptides can be derived from natural sources or synthesized in the laboratory [12,13].

In wound healing, it has been shown that they prevent or control infection by disrupting microbial membranes and modulate immune response by reducing excessive inflammation [14,15,16]. In addition to antimicrobial and anti-inflammatory properties, they also promote tissue regeneration [17]. Some peptides have been shown to promote angiogenesis that is crucial for wound healing, while others support migration and proliferation of keratinocytes, fibroblasts, and endothelial cells that are essential for re-epithelialization and tissue repair. Furthermore, peptides can promote collagen synthesis, which provides structural integrity to the new tissue [18].

Given the wound-healing promoting properties of certain peptides, this study aimed to investigate whether the transplantation of FTSCs combined with a regeneration promoting peptide hydrogel could enhance healing. Specifically, based on previous studies, we chose to test TSN6, a peptide identified following collagenase digestion of extracellular matrix (ECM) which is derived from the coiled-coil domain of multimerin-1 (MMRN1). The TSN6 has shown to promote tissue repair in a mouse wound healing model and increase keratinocyte, fibroblast, and endothelial proliferation in vitro [19,20]. In this study, the TSN6 peptide was incorporated into a carboxymethylcellulose (CMC) hydrogel and applied to porcine full-thickness wounds grafted with FTSCs and compared to controls. The effects on wound healing and quality of healing were evaluated.

## 2. Materials and Methods

### 2.1. Carboxymethylcellulose (CMC) Hydrogel

To prepare the carboxymethylcellulose (CMC) hydrogel, equal parts of low and high molecular weight CMC were blended in a 1:1 ratio. The CMC mixture was then gradually added to saline preheated to 70 °C to achieve a final concentration of 3% (*w*/*v*). The resulting hydrogel was homogenized using positive displacement pipettors.

### 2.2. Peptides

This study utilized peptide TSN6, with a scrambled version of the peptide serving as a control. The TSN6 sequence, HSPDIQLQKGLTFEPIQIK, is derived from the coiled-coil domain of multimerin-1 (MMRN1) [19,20]. TSN6 was synthesized either by the Tufts University Core Chemistry Facility or by ABI Scientific, Inc., with peptide identity and purity (>98%) confirmed by high-performance liquid chromatography (HPLC). Each stock peptide solution was prepared in phosphate buffer (0.015 M sodium phosphate). Peptides were first dissolved in cold water on ice, then diluted to the desired volume to achieve a final concentration of 2 mg/mL The stock solution was aliquoted into single-use vials based on the volume required to achieve a final peptide concentration of 0.1 mg/mL when mixed with the CMC hydrogel. The 0.1 mg/mL concentration of TSN6 was determined to be optimal based on previously published work involving cell-based screens and preclinical in vivo dose–response studies [20]. Aliquots of the concentrated peptide stock were stored at −80 °C until use. Peptides were subsequently incorporated into the CMC hydrogel using positive displacement pipettors.

### 2.3. Animals and Anesthesia

All animal procedures for this study were approved by the University of Texas Health Science Center at San Antonio (UTHSCSA), the Institutional Animal Care and Use Committee (IACUC, Protocol # 20220034AR), and the Animal Care and Use Review Office (ACURO, Protocol # USUHSFY22001.e001). Two anesthetized pathogen-free Yorkshire Hybrid pigs (Midwest Research, Glencoe, MN, USA) weighing between 50 and 70 kg were utilized for this study. The animals were individually housed at the UTHSCSA animal facility and acclimated for at least 72 h and fasted 8 h prior to planned procedures. Anesthesia and analgesia (buprenorphine SR-LAB (0.1–0.24 mg/kg)) were used for primary wound creation and anesthesia for dressing changes. Anesthesia was induced with an IM injection of tiletamine-zolazepam (Telazol, 4–6 mg/kg) or ketamine (10–25 mg/kg). Pigs were then intubated with an endotracheal tube and placed on a mechanical ventilator and anesthesia was maintained with 1–5% isoflurane. Heart rate, oxygen saturation, body temperature, muscular relaxation, and urine output were monitored during the procedure. After completion of the procedure, pigs were transferred to their respective holding rooms, observed twice per day for the first 72 h, and administered buprenorphine SR-LAB every 72 h or as needed based on post-surgical observations. Animals were followed for 14 or 28 days to examine wound healing progression and were euthanized under anesthesia according to institutional IACUC policies using a commercial euthanasia solution IM.

### 2.4. Study Design

**Figure 1:**
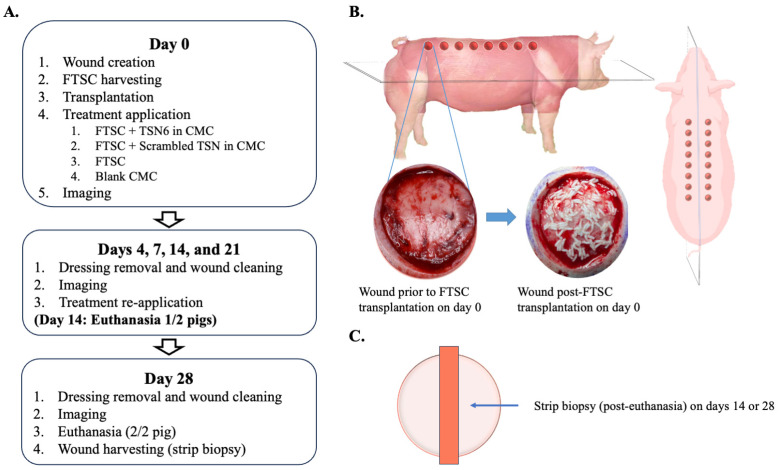
(**A**) Study design. (**B**) Wound locations and an example of a full-thickness skin column (FTSC) grafted wound. (**C**) Following euthanasia on days 14 or 28, a strip biopsy spanning the wound from edge to edge was collected for histological analysis.

A total of 32 standardized full-thickness excisional wounds (5 cm in diameter) were created on the dorsum of two anesthetized pigs (16 wounds per pig, with 8 on each side of the spine) using sterile scalpels. They were spaced ≈ 3 cm apart to minimize site-to-site interaction. Wounds were then randomized into four treatment groups (n = 4 per treatment per animal) using a randomized block design to minimize anatomical bias: (1) FTSC + TSN6, (2) FTSC + scrambled peptide, (3) FTSC alone, and (4) blank CMC hydrogel. The study design is depicted in Figure 1A,B.

Full-thickness skin columns (FTSCs) were harvested using 1.5 mm biopsy punches from multiple donor sites on the rump of the pig, at a density of up to eight 1.5 mm-diameter columns per cm^2^. The FTSCs were applied to the wound bed at an expansion ratio of 1:10. Hydrogels (up to 3 mL in volume) containing a novel peptide formulation and carboxymethylcellulose gel (3% *w*/*v*) were then placed on top. Following treatment application, wounds were covered with Tegaderm™. Secondary dressings consisted of Telfa™, Coban™, and a mesh SurgiVest jacket.

Wounds were assessed, imaged, and hydrogels reapplied on postoperative days 4, 7, 14, and 21 (Figure 1A). On day 14, following wound assessments, one of the two animals was euthanized, and excisional biopsies were collected from each wound for histological (Figure 1C). The remaining animal was monitored until day 28. This study design was chosen to allow harvesting of large strip biopsies, which provide a more comprehensive cross-section of the wound compared to small punch biopsies, especially in a study involving skin column transplantation.

### 2.5. Biopsies and Sample Processing

Full-thickness strip biopsies were harvested on days 14 and 28 immediately after euthanasia. Biopsies were surgically removed from wound edge-to-edge, including a small margin of uninjured tissue. Subsequently, the collected strip biopsies were fixed in 10% neutral-buffered formalin for at least 48 h, dehydrated, and embedded in paraffin for histological analysis. Sections 5–7 µm thick were cut using a microtome (Leica Biosystems, Buffalo Grove, IL, USA) and processed for histology with hematoxylin and eosin (H&E) and Masson’s trichrome stain (MTS). The stained sections were scanned using a Zeiss Axioscan Z1 (Zeiss, Jena, Germany) automated slide scanner and the ZEN 2.3 software (Zeiss).

### 2.6. Outcome Measures

All the analyses were performed in a blinded fashion.

#### 2.6.1. Histology

Re-epithelialization was assessed on days 14 and 28 using H&E- and MTS-stained wound strip sections. Wound and re-epithelialized tissue areas were measured by tracing with ImageJ 1.53K software (NIH, Bethesda, MD, USA). The percentage of re-epithelialization was calculated as the area of newly formed epithelium divided by the original wound area.

Neo-epidermal thickness on day 28 post-transplantation was measured from H&E- and MTS-stained wound strip sections at 10 representative areas of the newly formed epidermis in each wound cross-section and presented as a mean value.

The number of rete ridges in the neo-epidermis was assessed from day 28 H&E- and MTS-stained histological sections. Rete ridges were counted across the wound cross-section, divided by the wound length, and reported as rete ridges per millimeter (rete ridges/mm).

The presence of skin adnexal structures was quantified by counting the number of adnexal units (hair follicles and glands) in H&E- and MTS-stained sections on day 28 post-wounding. Inflammation was assessed on day 28 post-wounding based on the presence of inflammatory cells, including lymphocytes, macrophages, eosinophils, and neutrophils, using the following scale: 0 = none, 1 = minimal, 2 = mild, 3 = moderate, 4 = marked.

#### 2.6.2. Macroscopic Imaging

Macroscopic images were captured using the Silhouette Star imaging system (Aranz Medical Ltd., Christchurch, New Zealand) to assess wound closure and contraction. Wound closure was quantified as the area of the remaining open wound on days 7, 14, 21, and 28, expressed as a percentage of the original wound area on day 0. Wound contraction was evaluated on day 28 as the percentage reduction in wound area from day 0. The Antera 3D imaging device (Miravex, Dublin, Ireland) was used to capture 3-dimensional wound images on day 28. Collected images were analyzed and measured for pores [pore count/cm^2^] using the Antera 3D imaging software (Antera 3.x Software, Miravex).

### 2.7. Power and Statistical Analysis

A power analysis was performed using a one-way ANOVA with pairwise, two-sided equality comparisons. The analysis concluded that 4 to 8 samples per treatment group are sufficient to achieve 80% power with a type I error rate of 0.05. Statistical analysis was performed using GraphPad Prism (GraphPad Software 10.5.0, Inc., La Jolla, CA, USA). The Shapiro–Wilk test was used to assess data normality. Comparisons between treatment groups were made using unpaired one-way analysis of variance (ANOVA), followed by Tukey’s multiple comparisons test. *p*-values < 0.05 were considered statistically significant. Data are presented as mean ± SEM. In the porcine study, the sample size for each treatment group was n = 4–8, with two biological replicates.

## 3. Results

### 3.1. Wound Healing

Wound healing was evaluated by assessing wound closure on days 7, 14, 21, and 28, wound contraction from macroscopic images on day 28, and re-epithelialization in H&E- and MTS-stained sections on days 14 and 28. In addition, tissue regeneration was assessed by quantifying the number pores on the injured area on days 14, 21, and 28 post-wounding. Skin pores are small openings at the surface of the skin that represent outlets for hair follicles and glands. As such, pore presence can serve as a marker of tissue regeneration.

**Figure 2:**
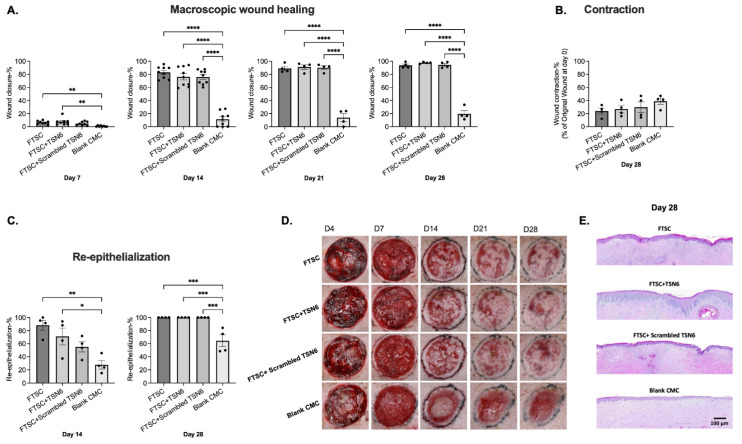
(**A**) Macroscopic wound healing: By day 14, transplanted wounds were over 75% closed, while the blank CMC group remained 90% open. By day 28, transplanted wounds were nearly fully closed (>94%), versus 20% in the blank group. Differences between transplanted and untransplanted wounds were significant on all days, with no differences among transplanted groups. (**B**) Wound contraction: No significant differences in wound contraction were observed among the groups on day 28 post-wounding. (**C**) Re-epithelialization: By day 14, wounds treated with FTSC and FTSC + TSN6 were significantly more re-epithelialized compared to the blank CMC-treated wounds. By day 28, all FTSC-treated wounds were fully re-epithelialized, with a statistically significant difference compared to ungrafted wounds. (**D**) Representative macroscopic images of the wounds over time. (**E**) Representative H&E images of the wounds on day 28 post-wounding. * 0.01 < *p* < 0.05, ** 0.001 < *p* < 0.01, *** 0.0001 < *p* < 0.001, **** *p* < 0.0001.

Macroscopic wound closure analysis showed that by day 7, wounds treated with FTSC, FTSC + TSN6, FTSC + scrambled TSN6, and blank CMC hydrogel were closed by 6 ± 1%, 8 ± 2%, 5 ± 1%, and 1 ± 0%, respectively. Wound closure in the FTSC and FTSC + TSN6 groups was significantly greater compared to the blank control (*p* ≤ 0.01). By day 14, all transplanted wounds were over 75% closed, while the blank CMC group remained just over 10% closed. By day 28, transplanted wounds were nearly fully closed (>94%), whereas the blank CMC group reached only 20% closure. On days 14, 21, and 28, the difference between transplanted and untransplanted wounds was statistically significant (*p* ≤ 0.001). No significant differences were observed between the transplanted groups (Figure 2A).

The overall percentage of wound contraction from day 0 to day 28 was determined from macroscopic images of each wound. The FTSC, FTSC + TSN6, FTSC + scrambled TSN6, and blank CMC hydrogel treatment groups exhibited wound contraction of 24 ± 4%, 27 ± 5%, 30 ± 8%, and 39 ± 5%, respectively. Contraction was observed in all wounds, with the blank CMC hydrogel group showing a trend towards the greatest contraction. However, no statistically significant differences were observed between treatment groups (Figure 2B).

The average percentage of wound re-epithelialization on day 14 post-wounding for the FTSC, FTSC + TSN6, FTSC + scrambled TSN6, and blank CMC hydrogel was 88 ± 8%, 71 ± 13%, 55 ± 8%, and 28 ± 6%, respectively. The FTSC (*p* ≤ 0.01)- and the FTSC + TSN6 (*p* ≤ 0.05)-treated wounds were statistically significantly more re-epithelialized than the blank CMC wounds (Figure 2A). By day 28, all the FTSC-treated wounds were fully re-epithelialized and the difference to the untreated wounds was statistically significant (*p* ≤ 0.001) (Figure 2C). Representative macroscopic and histological images of the wounds are shown in Figure 2D and E, respectively.

The number of pores in the healing wound area was measured on days 14, 21, and 28 using a 3D imaging system. On day 14, wounds treated with FTSC, FTSC + TSN6, FTSC + scrambled TSN6, and blank CMC hydrogel showed 117 ± 10, 108 ± 12, 99 ± 10, and 67 ± 7 pores, respectively. Wounds treated with FTSC and FTSC + TSN6 had significantly more pores than those treated with blank CMC hydrogel. However, no statistically significant differences were observed among treatment groups on days 21 and 28 (Supplemental Figure S1).

### 3.2. Donor Site Healing

The FTSC donor sites healed well and were closed by the first dressing change on day 4 post wounding.

### 3.3. Quality of Healing

The quality of healing was evaluated histologically on day 28 by measuring the thickness of the newly formed neo-epidermis and the number of rete ridges. In addition, inflammation in the healing wounds was assessed on day 28.

**Figure 3:**
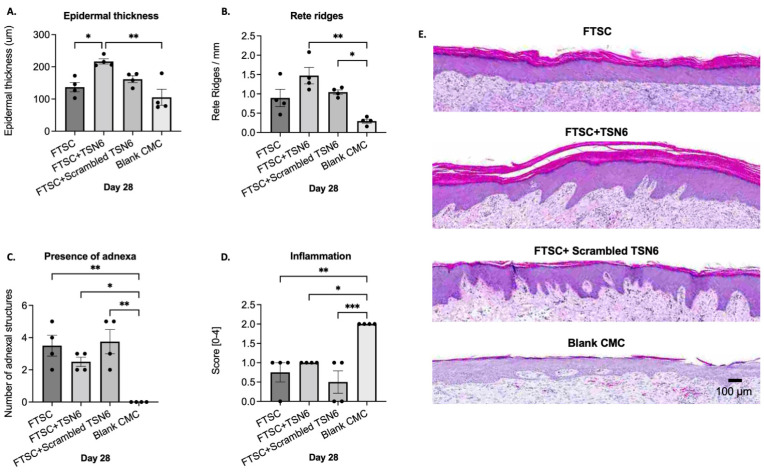
(**A**) Epidermal thickness: The FTSC + TSN6-treated wounds exhibited a statistically significantly thicker epidermis than the FTSC and the ungrafted groups. (**B**) Number of rete ridges: The FTSC + TSN6 and FTSC + scrambled TSN6 groups had more rete ridges than the other groups, with a statistically significant difference compared to the blank CMC hydrogel-treated wounds. (**C**) Presence of adnexa: All the FTSC transplanted wounds had significantly more adnexal structures than the blank CMC group. (**D**) Inflammation: All the transplanted groups demonstrated significantly less inflammation than the untransplanted blank CMC group. (**E**) Representative H&E-stained images of the wounds on day 28. * 0.01 < *p* < 0.05, ** 0.001 < *p* < 0.01, *** 0.0001 < *p* < 0.001.

Neo-epidermal thickness in the FTSC, FTSC + TSN6, FTSC + scrambled TSN6, and blank CMC hydrogel wounds was 137 ± 15 µm, 216 ± 8 µm, 161 ± 10 µm, and 105 ± 25 µm, respectively. The FTSC + TSN6 treated wounds produced a statistically significantly thicker epidermis than the FTSC (*p* ≤ 0.01) and the ungrafted (*p* ≤ 0.01) groups (Figure 3A).

The number of rete ridges/mm in the FTSC, FTSC + TSN6, FTSC + scrambled TSN6, and blank CMC hydrogel wounds was 0.9 ± 0.2, 1.5 ± 0.2, 1.0 ± 0.1, and 0.3 ± 0.1, respectively. The FTSC + TSN6 and the FTSC + scrambled TSN6 treated wounds had more rete ridges than the other wounds and the difference to the blank CMC hydrogel treated wounds was statistically significant (Figure 3B).

The number of skin adnexal units was quantified, and the results showed that, on average, the FTSC, FTSC + TSN6, FTSC + scrambled TSN6, and blank CMC hydrogel-treated wounds had 3.5 ± 0.6, 2.5 ± 0.3, 3.8 ± 0.8, and 0.0 ± 0.0 adnexal units, respectively. All transplanted groups had significantly more adnexal structures than the untransplanted control (*p* < 0.05), with no significant differences observed among the transplanted groups (Figure 3C).

The degree of inflammation was evaluated on post-wounding day 28 by analyzing and scoring the H&E-stained wound sections for infiltration of inflammatory cells to the wound bed. The results show that inflammation was minimal in the transplanted groups and mild in the blank CMC group. All the transplanted groups demonstrated significantly less inflammation (*p* < 0.05) than the untransplanted blank CMC group. No statistically significant differences between the transplanted groups were seen (Figure 3D). Representative histological images of the wounds are shown in Figure 3E.

## 4. Discussion

FTSC grafting, an alternative to the standard of care (STSG) technique, has been investigated in multiple preclinical and clinical studies, and shown to be a feasible approach [21]. Therapeutic peptides have also been extensively studied and their role in preventing infection and promoting wound healing has been well documented [22]. The purpose of this study was to evaluate whether a TSN6 peptide, mixed into a CMC hydrogel, could enhance the rate and quality of wound healing when applied to full-thickness wounds grafted with FTSC.

Our results showed that FTSC-grafted wounds were significantly more re-epithelialized on day 14 than the ungrafted wounds. By day 28 the grafted wounds were fully closed, whereas the ungrafted wounds remained approximately 40% open. However, no statistically significant differences were observed between the grafted groups, suggesting that in this model, the addition of TSN6 peptide did not further accelerate wound healing. It is important to consider that these were acute wounds created on young, healthy pigs, which tend to heal rapidly, especially when grafted with autologous skin. Therefore, observing no differences in healing speed is not uncommon. To better assess the therapeutic potential of TSN6, future studies could utilize models of impaired wound healing. Previously, Demidova-Rice et al. (2012) used similar endothelial ECM-derived peptides in a rodent model of impaired healing and showed that the treatment accelerated wound healing in comparison to controls [19]. Similarly, Sheets et al. (2016) investigated the effects of matrix- and plasma-derived peptides in a diabetic porcine model and demonstrated that peptide treatment significantly increased reepithelialization and angiogenesis, compared to saline-treated controls wounds [20].

Wound healing was also assessed by quantifying skin pores using a 3D camera. The reappearance of pores in the healing wound may indicate successful regeneration and restoration of normal skin architecture [23]. Our data showed significantly higher pore counts in the FTSC-grafted wounds compared to the ungrafted wounds on day 14. One advantage of FTSC grafting over STSG is that, being full-thickness, they include skin adnexal structures such as hair follicles and glands [24]. The presence of adnexal units on day 28 post-wounding was assessed histologically and all the FTSC-grafted wounds had significantly more adnexal structures than the untransplanted wounds. Similar findings were reported by Rettinger et al. (2017), who grafted full-thickness porcine wounds with FTSCs and observed enhanced re-epithelialization and the presence of adnexal structures compared to ungrafted controls [25]. In the present study, the TSN6 peptide did not further increase the number of adnexal units in FTSC-transplanted wounds. However, Xavier et al. (2025) demonstrated that TSN6 significantly increased the number of hair follicles in a murine wound healing model [26].

In addition to wound healing, the effect of the TSN6 peptide on quality of healing was assessed on post-operative day 28, using histology and macroscopically with different non-invasive imaging systems. Neo-epidermal thickness and number of rete ridges, that are both important indicators of skin regeneration following wound healing, were measured using H&E- and MTS-stained slides. Our results showed that the TSN6 peptide increased epidermal thickness and the number of rete ridges in the newly formed epidermis in comparison to the other groups. This is important, since a thicker, more structured neo-epidermis suggests a more mature and functionally restored epidermal layer once the wound has re-epithelialized, whereas a thinner neo-epidermis may indicate incomplete healing [27,28,29]. Furthermore, the presence of rete ridges increases the structural integrity of the skin by strengthening the dermal–epidermal junction and improving resistance to mechanical stress [30]. Conversely, the lack of rete ridges is a characteristic of scar tissue lacking the mechanical and physiological properties of normal skin [31]. Previous studies support these findings. Similarly to the present study, Xavier et al. (2025) demonstrated increased epidermal thickness in murine full-thickness wounds following treatment with TSN6 [26]. In addition, Lin et al. (2021) treated full-thickness excisional wounds with collagen-derived peptides and showed that the treatment enhanced the quality of healing, as evidenced by the formation of a thicker neo-epidermis compared to controls [32]. In another mouse study, Wang et al. (2022) also demonstrated increased epidermal thickness after a treatment with an antimicrobial peptide cathelicidin-DM [33].

The mechanism by which peptides enhance neo-epidermal maturation during wound healing is not fully understood. However, TSN6 is a 19-amino acid synthetic peptide with partial sequence homology to the coiled-coil domain of multimerin-1 (MMRN1), an ECM-associated glycoprotein. It contains an N-terminal RGD motif, which may promote cell adhesion and tissue remodeling. TSN6 may also recapitulate MMRN1 signaling via receptors such as HMMR and CDK1 or function similarly to matrikines that are ECM-derived peptides promoting healing by modulating proliferation and growth factor signaling. Whether TSN6 mimics MMRN1 or acts through related ECM-receptor pathways remains under investigation [26].

Wound contraction, an important indicator of quality healing, was evaluated macroscopically. Although an essential part of healing, excessive contraction can compromise healing through induction of fibrosis leading to development of wound contractures limiting elasticity and function [34,35]. It is well known that grafted wounds contract less than ungrafted wounds, which was also observed in our study, although the differences between groups were not statistically significant [33]. Keenan et al. (2023) reported similar percent contraction on day 28 following FTSC transplantation [36]. In this study, we did not find that the TSN6 would have any effect on wound contraction. Previous studies in rodent models have demonstrated that Sipunculus nudus- and Lates calcarifer-derived peptides increase wound contraction [32,37]. While enhanced contraction may be interpreted as beneficial in rodent models, it is unclear how these results would translate to human or porcine wound healing.

Hyperpigmentation, a common outcome after wound healing, particularly in deeper injuries, was also evaluated using a 3D camera. Our results demonstrated significantly less hyperpigmentation in the FTSC-grafted wounds. This is consistent with previous studies showing that full-thickness skin grafts result in fewer pigmentation changes compared to STSG [38,39,40,41]. In this study, TSN6 treatment did not appear to influence pigmentation.

## 5. Conclusions

This preclinical study in pigs investigated whether transplantation of FTSCs combined with the endothelial ECM peptide TSN6 could enhance wound healing outcomes. The results showed no significant effect on the rate of wound healing in this acute wound model. However, it was shown that the TSN6 mixed in a CMC hydrogel significantly enhanced quality of healing. Since the sample size includes only two biological replicates, the results should be considered preliminary. Future studies with larger sample sizes, longer follow-up times, and models of impaired wound healing are needed to further evaluate and validate these preliminary findings.

## Data Availability

The raw data supporting the conclusions of this article will be made available by the authors on request.

## Supplementary Materials

The following supporting information can be downloaded at: https://www.mdpi.com/article/10.3390/bioengineering12080856/s1, Figure S1: A. Pores on skin: Wounds treated with FTSC and FTSC + TSN6 showed significantly higher pore counts compared to those treated with blank CMC hydrogel. No statistically significant differences among the treatment groups were observed on days 21 and 28. B. Representative 3D images of the wounds on day 14.

## Abbreviations

Analysis of variance	ANOVA
Animal Care and Use Review Office	ACURO
Carboxymethylcellulose	CMC
Extracellular matrix	ECM
Full-thickness skin column	FTSC
Hematoxylin and eosin	H&E
Institutional Animal Care and Use Committee	IACUC
Masson’s trichrome stain	MTS
Multimerin-1	MMRN1
Split-thickness skin grafting	STSG
Translin	TNS
University of Texas Health Science Center-San Antonio	UTHSCSA
