# Supplementary material for: The Use of Therapeutic Peptides in Combination with Full-Thickness Skin Columns to Improve Healing of Excisional Wounds
Source: Bioengineering (Basel). 2025 Aug 9;12(8):856. doi: 10.3390/bioengineering12080856 (PMC12383989; doi:10.3390/bioengineering12080856)
Supplement: Supplementary file 1 [file bioengineering-12-00856-s001.zip › bioengineering-3796991-supplementary.pdf]

Article

The Use of Therapeutic Peptides in Combination with Full-Thickness Skin Columns to Improve Healing of Excisional Wounds

Anders H. Carlsson [1], Ira M. Herman [2,3,4], Sean Christy [5], David Larson [6], Rodney K. Chan [1], Thomas N. Darling [7] and Kristo Nuutila [5,*]

1 Metis Foundation, San Antonio, TX 78216, USA; carlsson@metisfoundationusa.org (A.H.C.); rodneykchan@gmail.com (R.K.C.)
2 Department of Developmental, Molecular, and Chemical Biology, Tufts University School of Medicine, Boston, MA 02111, USA; ira.herman@tufts.edu
3 Center for Innovations in Wound Healing Research, Tufts University School of Medicine, Boston, MA 02111, USA
4 Tissue Health Plus, Inc., Fort Worth, TX 76102, USA
5 United States Army Institute of Surgical Research, Joint Base San Antonio-Fort Sam Houston, San Antonio, TX 78234, USA; sean.e.christy.ctr@health.mil
6 The Department of Surgery, University of Texas Health, San Antonio, TX 78229, USA; larsond2@uthscsa.edu
7 Department of Dermatology, Uniformed Services University, Bethesda, MD 20814, USA; thomas.darling@usuhs.edu
* Correspondence: kristo.j.nuutila.ctr@health.mil

Supplementary Materials

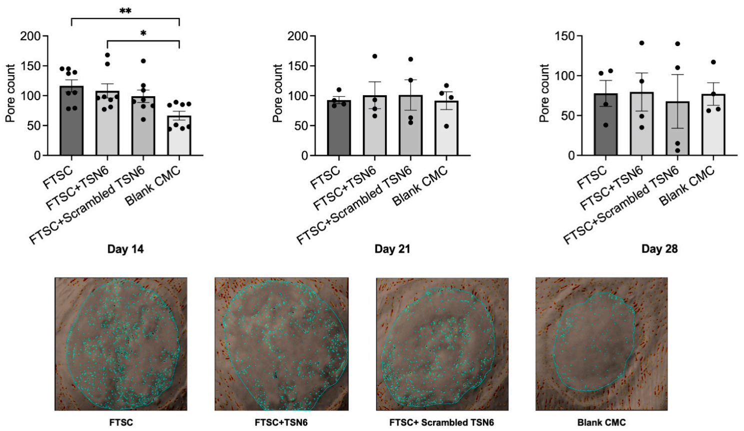

Supplemental Figure S1. A. Pores on skin: Wounds treated with FTSC and FTSC + TSN6 showed significantly higher pore counts compared to those treated with blank CMC hydrogel. No statistically significant differences among the treatment groups were observed on days 21 and 28. B. Representative 3D images of the wounds on day 14.
